# Mullerian-Type Ciliated Cyst of the Thigh with PAX-8 and WT1 Positivity: A Case Report and Review of the Literature

**DOI:** 10.1155/2016/2487820

**Published:** 2016-12-14

**Authors:** Corinthia Fabien-Dupuis, Brian Cooper, Jeffrey Upperman, Shengmei Zhou, Nick Shillingford

**Affiliations:** ^1^Department of Pathology and Laboratory Medicine, Children's Hospital Los Angeles, Los Angeles, CA, USA; ^2^Keck School of Medicine of the University of Southern California, Los Angeles, CA, USA; ^3^Department of Pathology, Queen Elizabeth Hospital, Bridgetown, Barbados; ^4^Department of Surgery, Children's Hospital Los Angeles, Los Angeles, CA, USA

## Abstract

Mullerian-type ciliated cysts are uncommon lesions usually found in the lower extremities and perineal region of young females. They have however been reported in males and in other anatomic sites. The cyst lining is typically positive for estrogen receptor (ER), progesterone receptor (PR), PAX-8, and WT1 immunohistochemical stains. This staining pattern has led to the notion that these cysts are of Müllerian origin. The vast majority of cases are located in the dermis where the preferred nomenclature is cutaneous ciliated cyst (CCC). We report a case of Müllerian-type ciliated cyst in the thigh of a 16-year-old girl. Unlike most of the cases reported in the English literature, this cyst was not centered in the dermis. Only a few other cases of Müllerian-type ciliated cysts with no cutaneous connection have been reported. We propose the term ectopic Müllerian cyst for this rare subset of lesions that are not skin based as is the current case.

## 1. Introduction

Cutaneous ciliated cysts (CCC) also known as cutaneous Mullerian cysts are rare lesions which mostly occur in the lower extremities of females [1]. Up to 2015, 60 cases of CCC had been published, of which 50 were in female patients and 10 in males. These cysts are believed to arise from ectopic Mullerian rests which become active during puberty or pregnancy [2]. We present a case of a CCC without obvious cutaneous connection occurring in the thigh of a 16-year-old girl [3]. By immunohistochemistry, estrogen receptor (ER), progesterone receptor (PR), PAX-8, and WT1 were positive in the epithelial lining of the cyst, lending support to a possible Müllerian origin [4].

## 2. Case Report

A 16-year-old girl presented with a solitary, 2 cm, freely mobile mass in the soft tissue of the right thigh. The cyst had been present for at least 3 years. According to the patient, the mass has waxed and waned but was for the most part increasing in size. There is no associated rubor, calor, pain, tenderness, skin changes, or drainage. She denied fever, anorexia, asthenia, and weight loss. She has no significant past medical or surgical history. There is no history of pregnancy or preceding trauma. At surgery, the mass was situated in the subcutaneous soft tissue. It was incised revealing its cystic nature. The cystic fluid was expressed and the mass was completely excised and sent to the pathology department for further evaluation.

**Figure 1:**
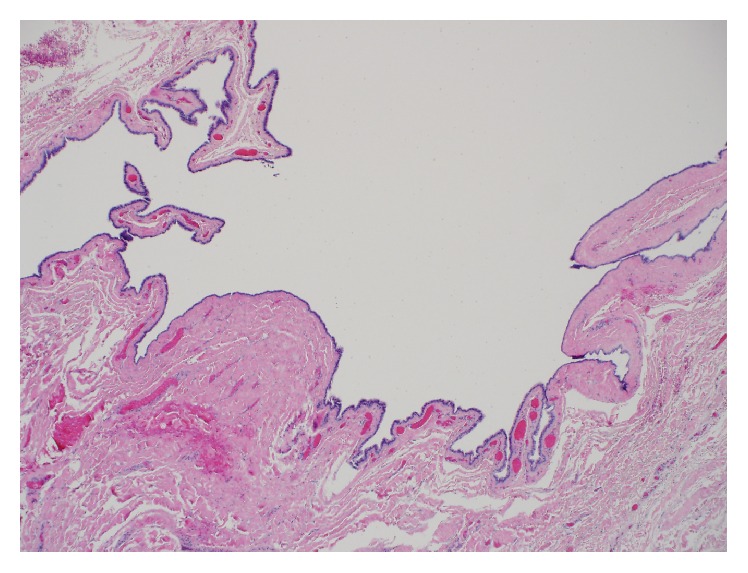
Low power photomicrograph showing a cystic mass with infoldings and excrescences of its wall at the inner aspect. The cyst lining is easily appreciated. H&E. Magnification, 40x.

**Figure 2:**
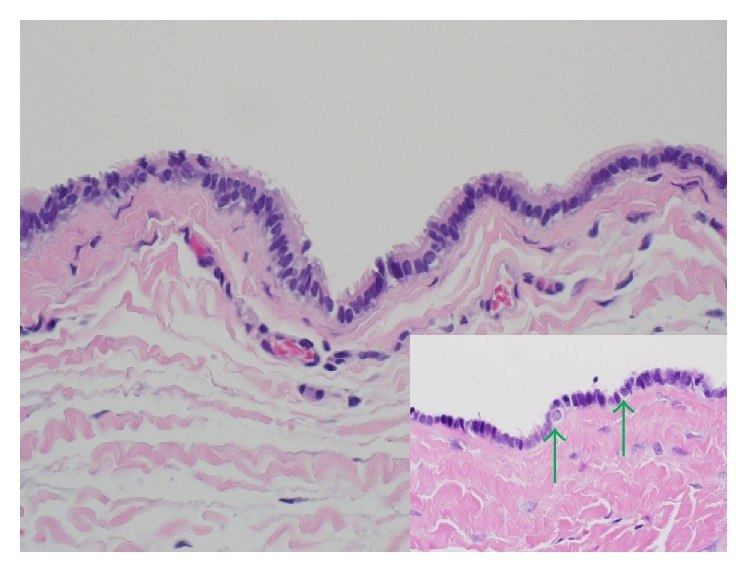
Higher magnification reveals that the cyst is lined by a ciliated simple columnar epithelium reminiscent of that of Mullerian derived structures. H&E. Magnification, 400x. The inset highlights occasional cells with round nuclei and perinuclear clearing at the base of the epithelium (green arrows). These cells are similar to those seen in fallopian tube epithelium. H&E. Magnification, 600x.

**Figure 3:**
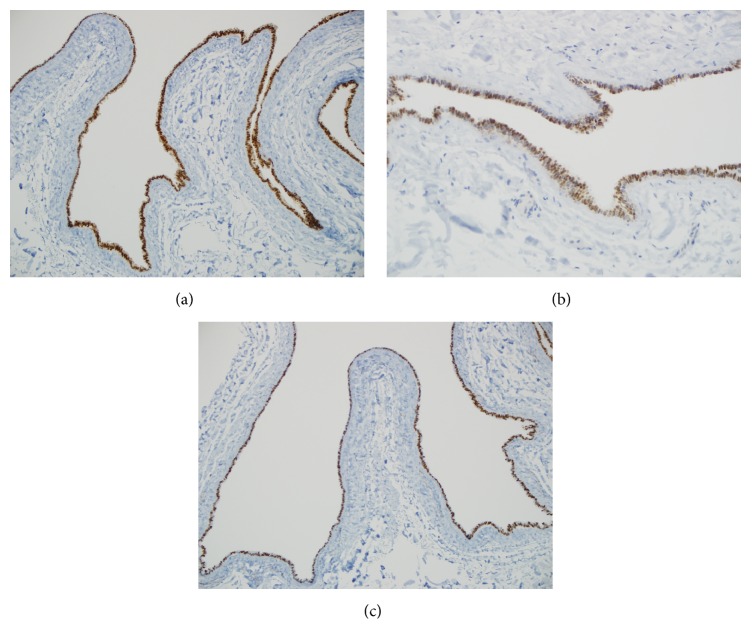
The immunohistochemical staining profile supports the notion of a Mullerian origin. Progesterone receptor immunostain shows strong and diffuse positivity in the cyst lining epithelium ((a) magnification, 100x) and PAX-8 is positive in the majority of the epithelial lining cells ((b) magnification, 200x). The lining is strongly and diffusely positive for WT1 immunostain which is localized to the nuclei ((c) magnification, 100x).

Surgical pathology received a 2.5 cm tan-brown collapsed unilocular cyst. Skin was not identified in the specimen on gross inspection. The cyst wall measured 0.1 cm in thickness. The inner lining was tan, smooth, and glistening. Representative sections were submitted for microscopic examination. On low magnification, sections showed a cystic lesion whose fibrocollagenous wall was thrown into multiple folds forming finger-like projections focally (Figure 1). A thorough search for skin appendages such as hair follicles, sebaceous glands, and sweat glands revealed no such structures. High magnification revealed a cyst lining formed by a* simple* ciliated cuboidal to columnar epithelium with no significant cytologic atypia (Figure 2). Pseudostratification was evidenced focally. Occasional cells with round nuclei and perinuclear clearing seen towards the base of the epithelium were reminiscent of cells seen in fallopian tube epithelium (Figure 2, inset). In the fallopian tubes these cells are thought to be intraepithelial lymphocytes. No smooth muscle, cartilage, mucus glands, or adnexa were identified in the cyst wall. There was no significant inflammation. By immunohistochemistry, the epithelial cells were positive for pan-cytokeratin (AE1/AE3), estrogen receptor (ER), progesterone receptor (PR), PAX-8, and WT1 (Figures 3(a), 3(b), and 3(c)). The latter 4 are markers of Mullerian origin.

## 3. Discussion

Cutaneous ciliated cyst is an uncommon lesion with only 60 cases reported in the English literature between 1890 and 2015 (Table 1). The entity was first described by Hess in the seminal paper in 1890 [5]. The term cutaneous ciliated cyst was eventually coined by Farmer and Helwig in 1978 to describe a unique type of cyst arising in the lower extremities of young women. They reported lesions occurring in 11 patients, all of whom were female, with an age range from 15 to 30 years. The average age was 22. In their theory, they proposed that heterotopic Mullerian tissue is sequestered during embryonic development. This results in hormone responsive Mullerian rests being deposited at specific sites leading to the formation of Mullerian-type cysts after puberty when there is an increase in hormone production. These cysts may show a growth phase during pregnancy when hormonal activity is once again elevated [1, 4, 6]. The origin still remains controversial today with various theories being proposed. These theories range from the previously mentioned Mullerian heterotopia to metaplasia of sweat gland (eccrine) epithelium to embryonic remnants of the cloacal membrane [5, 6].

Mullerian cysts may present as solitary unilocular or multilocular lesions. The ciliated epithelial lining is reminiscent fallopian tube epithelium [5]; however squamous metaplasia may be present focally [1]. There is generally no cytologic atypia or increased mitotic activity. The majority of cysts being located in the lower limbs may be explained by the close proximity of the Mullerian ducts to the lower limbs during development. However, the presence of similar cysts at distant sites such as scalp and mediastinum may be explained by vascular or lymphatic dissemination [7].

Despite the various theories proposed with respect to their origin, the Mullerian heterotopia theory is supported by this striking resemblance to fallopian tube epithelium and by the consistent nuclear positivity for antibodies to the steroid receptors for estrogen and progesterone [6]. PAX-8, a member of the paired box (PAX) family of transcription factors which is important in the development of Mullerian and thyroid organs, has been shown to be expressed in the nuclei of the lining cells of these ciliated cysts by immunohistochemistry in recent publications [4]. This finding further supports the possibility of a Mullerian origin as PAX-8 immunohistochemical stain is currently used to identify Mullerian tumors among others. Immunostain for WT1, the product of a gene which is essential for the development of the kidneys and gonads, is also positive lending further support to a Mullerian origin of these cysts. As a matter of fact the staining pattern of the epithelium for dynein is reported to be similar to that of fallopian tubal epithelium [8].

Of note however, similar cysts have been reported in male patients [6, 9, 10] and interestingly, at least 2 of these cases were from the scrotum [11, 12]. The presence of cysts with similar histomorphology in male patients has led some authors to gravitate towards the sweat gland origin theory as estrogen stimulation is thought to influence the development of Mullerian derived cysts. Leonforte described the first case of ciliated cyst in a male patient in 1982 and even then he noted histologic similarities to sweat glands. In his case he described the presence of PAS-positive granules and apical caps in the epithelial cells, both features of apocrine sweat glands. The presence of dilated eccrine ducts in the vicinity of the cyst and the fact that eccrine glands are normally present in the heel, the site of the cyst in his report, and a site devoid of apocrine glands led him to suggest a possible eccrine origin. The changes in the epithelium to resemble that of fallopian tube were attributed to a metaplastic process caused by inflammation and subsequent irritation of pluripotent cells triggered by the cyst's contents [13].

In 1997 Sidoni and Bucciarelli reported a case of a ciliated cyst in the skin of the perineum of a 60-year-old man, the origin of which they suggested was primitive caudal gut. They hypothesized that the cyst was derived from embryonic remnants of the cloacal membrane [14]. Cysts lined by nonsquamous epithelium in the perianal/perirectal region are thought to be the consequence of abnormal organogenesis of the caudal end of the embryo. The tailgut, the distalmost intestinal segment, along with the embryonic tail within which it lies undergoes complete physiologic atrophy at the 8 mm stage. Subsequently, the cloaca and cloacal membrane are divided by the urorectal septum giving rise to the anteriorly situated urogenital sinus and the posteriorly situated rectum. Incomplete tailgut atrophy may lead to sequestration of ectodermal and endodermal tissues into the soft tissue of the perianal and perineal regions leading to the formation for ciliated cysts [14].

It may be that these differing theories are substantial and that ciliated cysts of the skin and soft tissue form part of a heterogenous group of lesions developing from different histologic and embryonic structures. Hung et al. have proposed that the term cutaneous ciliated cyst be abandoned for “cutaneous Mullerian cysts” for those cysts which show features of Mullerian origin. They also propose the use of cutaneous ciliated eccrine cysts for the other group of cysts which occur in males and appear to be derived from ciliated metaplasia occurring in eccrine cells. Bivin Jr. et al. compared eccrine sweat glands with CCC histologically, immunohistochemically, and ultrastructurally and found them “completely unrelated” [15].

In this instance, the cyst was located in the upper thigh and showed strong staining not only for ER and PR but also for PAX-8 and WT1. These findings strongly support a Mullerian origin as mentioned by previous authors. Interestingly, unlike most cases of cutaneous ciliated cysts reported in the literature, our case showed no definitive evidence of cutaneous connection. A previously reported case with no attachment to skin was found in a postmenopausal woman on hormone replacement therapy with estrogen and gestagen [3]. We suggest the term ectopic Mullerian cyst be substituted for these rare lesions, eliminating the word cutaneous since a subset of these cysts is not related to the skin, as shown by the current case.

## Figures and Tables

**Table 1:** List of previously reported cases of ciliated cutaneous cysts from 1890 to 2015. A total of 60 cases were reported, of which 50 were in female patients and 10 in males. Forty-eight (48) of these lesions were located in the lower extremity and its vicinity including the inguinal region, scrotum, perineum, buttock, gluteal cleft, and sacrococcygeal region. The other locations included the back, neck, scalp, cheek, abdominal wall, umbilicus, paravertebral region, and thumb. The reported ages ranged from 7 to 60.

Number	Year	Sex	Age	Location	Duration	Authors
1	1890	Female	15	Back	Recent	Hess
2	1969	Female	39	Left leg	>10 years	Clark
3–6	1970	Female	15–27	Thigh × 2	2 to several years	Butterworth et al.
		Female		Right buttock		
		Female		Knee		
7–17	1978	Female	15–30	Buttock	Weeks to 5 years	Farmer, Helwig
		Female		Thigh × 4		
		Female		Calf × 4		
		Female		Foot × 2		
18	1980	Female	33	Right sole	2 months	True, Golitz
19	1982	Female	15	Thigh	7 months	Park et al.
20	1982	Male	42	Heel	5 years	Leonforte et al.
21	1983	Female	36	Left foot	Early teens	Ross, Schwartz
22	1990	Female	42	Right buttock	Long standing	Al-Nafussi, Carder
23	1990	Female	18	Left buttock	4 years	Varma et al.
24	1993	Female	22	Scapula	Long standing	Sabourin et al.
25	1994	Female	20	Scalp	3 years	Sickel et al.
26	1994	Male	28	Left foot	Not stated	Trotter et al.
27	1995	Female	16	Left buttock	Recent	Biernat, Biernat
28	1995	Female	14	Right thigh	10 months	Cortes-Franco et al.
29	1995	Female	19	Right buttock	2 years	Tachibana et al.
30	1995	Female	17	Right sole	2 years	Osada et al.
31	1995	Male	27	Right sole	2 years	Ashton
32	1997	Male	60	Perineal area	2 years	Sidoni et al.
33	1997	Female	11	Left foot	1 year	Innocenzia et al.
34	1999	Female	23	Right lower leg	1 year	Yokozaki et al.
35	2000	Female	12	Sacrococcygeal	Months	Dini et al.
36	2001	Female	13	Sacrococcygeal	1 year	Lee et al.
37	2002	Female	14	Lower abdomen	3 months	Fontaine et al.
38	2002	Male	53	Right cheek	2 years	Ohba et al.
39	2002	Female	18	Abdomen	6-7 months	Vadmal et al.
40	2004	Male	54	Perineum	New discovery	Santos et al.
41	2006	Male	56	Right inguinal area	3 years	Lee et al.
42	2006	Female	41	Umbilicus	3 months	Kim et al.
43	2007	Female	16	Left thigh	1 year	Chong et al.
44	2008	Female	54	Paravertebral	8 years	Businger et al.
45	2008	Male	15	Scrotal area	Recent	Pérez Valcárcel et al.
46	2009	Female	51	Left leg	2 years	Torisu-Itakura
47	2010	Female	13	Right leg	2 years	Bivin et al.
48	2011	Female	25	Not stated	2 years	Gelincik
49	2011	Female	48	Right heel	Several years	Stevens, Sarma
50	2011	Female	14	Right knee	4 years	Ashturkar et al.
51	2012	Female	16	Left thumb	Not stated	Hung et al.
52	2013	Female	15	Right hip	1 year	Rodrigo-Nicolás et al.
53	2014	Female	20	Left knee	10 years	Joehlin-Price et al.
54	2014	Female	22	Pretibial	2-3 years	Joehlin-Price et al.
55	2014	Female	13	Gluteal cleft	1 month	Oh et al.
56	2014	Female	53	Scalp	Lifelong	Reserva et al.
57	2014	Female	38	Popliteal fossa	1 year	Kavishwar et al.
58	2015	Male	14	Scrotum	Not stated	Swarbrick et al.
59	2015	Male	7	Left posterior neck	3 years	Kim, Kim
60	2015	Female	14	Right lower leg	1 year	Keisling et al.
